# Domestic violence perpetration, victimisation and self-poisoning in Sri Lanka: a protocol for a hospital-based case-control study

**DOI:** 10.1136/bmjopen-2024-089913

**Published:** 2025-04-03

**Authors:** Dilini Vidusha Hewa Kankanamge, Bruna Rubbo, Karen Morgan, Helen Cramer, Sharon Collard, Ramya Ekanayaka, Afra Latheef, Anushika Athapaththu, Mushfira Siddeek, Imalsha Wickramasuriya, Duleeka W Knipe, Thilini Rajapakse

**Affiliations:** 1 Department of Psychiatry, Faculty of Medicine, University of Peradeniya, Peradeniya, Central, Sri Lanka; 2 South Asian Clinical Research, Faculty of Medicine, University of Peradeniya, Peradeniya, Sri Lanka; 3 University of Bristol Medical School, Bristol, UK; 4 School of Social and Community Medicine, University of Bristol Medical School, Bristol, UK

**Keywords:** Suicide & self-harm, Case-Control Studies, MENTAL HEALTH

## Abstract

**Introduction:**

Domestic violence is a key risk factor for suicidal behaviour. While there is some evidence on the association between suicide and victimisation, there is a notable paucity of evidence pertaining to the perpetration of domestic violence and its association with suicidal behaviour. The aim of this study is to investigate the association between domestic violence (victimisation and perpetration) and self-poisoning in Sri Lanka.

**Methods and analysis:**

This is a hospital-based case-control study. Cases (n=260) will be individuals admitted to the toxicology ward of the Teaching Hospital Peradeniya, Sri Lanka, for medical management of self-poisoning. We will recruit controls (n=520) from other patients with unrelated conditions or accompanying visitors presenting to the outpatient department of Teaching Hospital Peradeniya. We will use unconditional logistic regression models to investigate the association between domestic violence and self-poisoning.

**Ethics and dissemination:**

We obtained ethics approval from the Ethical Review Committee of the Faculty of Medicine, University of Peradeniya, Sri Lanka. The research assistants will be trained in administering the questionnaire and ensuring participant safety. Results will be disseminated in peer-reviewed articles, local media and at national and international conferences.

**STRENGTHS AND LIMITATIONS OF THIS STUDY**

- This study focuses only on hospitalisations following self-poisoning, the predominant form of hospital-presenting self-harm in Sri Lanka.
- Hospital-control outpatients may differ from the general population in exposure distribution, such as higher rates of mood disorders and suicidal ideation, potentially introducing selection bias.
- The reported rate of domestic violence (victimisation and perpetration) may be underestimated owing to cultural stigma and recall bias.

## Introduction

 Suicide is an important public health issue globally. According to the WHO, more than 700 000 deaths occur owing to suicide every year.^1^ Among global suicide deaths, 77% occur in low and middle-income countries.^1^

Sri Lanka had one of the highest suicide rates in the world in 1995, with a rate of 47 per 100 000 population.^2^ Since then, the overall suicide rate has fallen, and in 2019, the suicide mortality rate in Sri Lanka was 14 per 100 000 population,^2^ which is 1.5 times the global average. However, rates of self-poisoning in Sri Lanka are still high, as in most areas of the world.^3^

Deaths owing to suicide in Sri Lanka are more common in males than in females, with a male to female suicide ratio of 4:1.^3^

However, self-harm is more common among females, most likely owing to several gender-related risk factors, including differences in the lethality of methods used, socially constructed differences such as gender roles, responsibilities and social status.^4^ Furthermore, pregnancy, miscarriages and postpartum periods are events unique to females which can affect suicidality.^5^

Women and girls are also more likely to be victims of domestic violence, which is known to be associated with increased risk of self-harm, both internationally and in Sri Lanka.^6 7^ Domestic violence refers to physical, sexual or emotional abuse and controlling behaviours (ie, physical, sexual, economic, emotional or psychological actions or threats of actions that can influence another person) usually by a current or former partner, but also by a family member or carer. Women are significantly more likely to be victims of domestic violence than men and are more likely to be injured because of it.^6^ Being a victim of domestic violence contributes to feelings of helplessness and vulnerability and increases the risk of self-harm and suicide.^8^ While domestic violence victimisation is more common in women, being a victim of domestic violence increases the risk of self-harm in both sexes.^9^ In the context of domestic violence and suicide, the COVID-19 pandemic may have introduced additional stressors which could impact individuals.^10^

While the association between domestic violence victimisation and self-harm and suicide has received some, although limited, attention, the association between domestic violence perpetration and self-harm and suicide has received even less. Previous research has shown an elevated risk of suicide in perpetrators of domestic violence,^11^ but this research has focused on high-income countries and evidence from low- and middle-income countries, like Sri Lanka, is lacking. The aim of this study is to explore the relationship between domestic violence (victimisation and perpetration) and self-poisoning in both sexes in Sri Lanka. The increased stigma surrounding disclosure for male victims arises from its conflict with traditional notions of masculinity.^12^

Hypothesis—there is a positive association between experiences of domestic violence and self-harm. Specifically, individuals who have been subjected to or perpetrated domestic violence are hypothesised to have a higher risk of self-harm due to the psychological and emotional distress caused by such trauma (victims) or due to perceived victimhood or loss of control (perpetrators).

## Methods and analysis

### Study setting

The study will be carried out at Teaching Hospital Peradeniya (THP), located in the Kandy District, Central Province of Sri Lanka. The Kandy District has a total population of 1 375 382 and is 115 km east from Colombo, the capital of Sri Lanka. Teaching Hospital Peradeniya is a tertiary referral hospital with a catchment area that includes the North Central, North Western and Sabaragamuwa Provinces. According to the 2019 Well Women Survey, the prevalence of domestic violence in the Kandy district is 15% and it is 17.4% in Sri Lanka.^13^

### Patient and public involvement

The questionnaire to be used in this study has been piloted by research assistants, local to the area, with patients in the toxicology unit and outpatient department of THP. Piloting highlighted that the original questionnaire was too long; therefore, it was shortened and simplified.

A workshop will be conducted involving community members aged 18 years and over in order to discuss the emerging survey findings and identify factors associated with domestic violence and to discuss associations of domestic violence with self-harm such as alcohol and other drug use, financial burden and depression.

### Study design

This study is a hospital-based case-control study. Cases will consist of both male and female patients admitted for medical management of self-poisoning to the toxicology unit in THP. All patients who present to THP owing to self-poisoning are admitted to the toxicology unit for observation and treatment, regardless of the severity of poisoning. We aim to recruit a consecutive series of cases. This case definition excludes self-harm owing to other methods, for example, hanging and cutting; however, self-poisoning represents the most common method of self-harm cases presenting to hospitals in Sri Lanka.^14^

Controls will consist of males and females, recruited from either other patients attending the outpatient department of THP for unrelated conditions or people accompanying other patients presenting at the outpatient department and clinic of the same hospital during the study period. These outpatients frequently present with conditions such as respiratory infection, diabetes, hypertension, pregnancy and conditions unrelated to the outcome of interest in this study. Controls will not be age or sex matched to cases, but we aim to recruit two controls for each case.

### Inclusion and exclusion criteria

All patients aged 18 years and above who have been admitted to the toxicology unit for medical management of self-poisoning will be eligible for inclusion as cases for the study. We will not include patients admitted for management of accidental poisoning. Accidental poisoning will be identified initially from the patient’s admission record and then verbally confirmed by the patient via self-report to the data collectors.

Controls will be either other patients aged 18 years and above attending the outpatient department of THP for unrelated conditions or accompanying visitors aged 18 years or above presenting at the outpatient department and clinic of the same hospital during the study period. Cases and controls who are physically unable or too ill to participate, as well as those who have been diagnosed with an intellectual disability or dementia, will be excluded from the study.

### Sample size

Based on a study previously conducted in this hospital, we estimated roughly 50 cases will be admitted for self-poisoning each month, and 50% of those will be male.^15^ Therefore, it was estimated that there will be 300 cases over a 6-month period. Based on a recruitment rate of 87%, reported in a previous study, we plan to collect data from 260 cases and 520 controls over 6 months.^16^ Assuming 20% of controls (odds=0.25) report having experienced domestic violence, we would be able to detect a twofold difference in risk with 82% power (alpha=0.05).^16^

### Data collection

After treatment, prior to discharge from hospital, eligible patients will be approached and informed about the study. The study’s purpose and nature will be explained, and participants will also receive an information leaflet. Participants who give informed written consent will be included. Data will be collected via face-to-face interviews, where research assistants will administer a questionnaire in a confidential space, ensuring privacy.

All cases and controls will be interviewed by a trained data collector. A psychiatrist (DVHK) will train and supervise four data collectors on the interview schedule, how to promote a safe environment for disclosure, building rapport with study participants and the actions to be taken when at-risk participants are identified. The baseline training of data collectors will be conducted in eight sessions, over 2 weeks. Lectures, discussions and role-play will be used in the training process.

We will use a prepiloted questionnaire, designed for the study, to conduct interviews in the participant’s preferred language (ie, Sinhala, Tamil or English) to collect data on suicidal behaviour, economic stressors, gambling, drug use, childhood adversity, psychiatric morbidity, alcohol use and domestic violence.

We will adapt questions from the domestic violence measure (‘impact tool kit’) to ask about domestic violence.^17^ Individuals’ financial status data will be captured by a culturally modified ‘Debit and finance survey’ questionnaire.^18^ Both of the above measures were shortened and piloted.

Data on psychiatric morbidity will be collected using the 9-item Patient Health Questionnaire (PHQ-9), which is a brief, one-page self-administered questionnaire that is internationally validated for the identification of depression and has been validated for use in Sri Lanka.^19^

The alcohol use disorders identification test (AUDIT) will be used to assess the alcohol intake behaviour of the participants. It has been validated for the local population.^20^

Data will be collected during non-visiting hours for patients in the toxicology unit to ensure responses are not influenced by another person and for patient safety. Identification details such as the name or the bed head ticket number (ie, the number of the file that contains the patient’s clinical updates) and date of birth will not be recorded. The questionnaire will first be completed in a paper form, and the data will then be recorded into an Access database. The lead researcher will conduct regular supervision of the entered data, check data entry for errors and shadow researchers to ensure consistency in approach.

During the data collection, if a participant becomes distressed, is found to be depressed or reports suicidal thoughts in the preceding 2 weeks, a referral to the doctor on call for the psychiatry unit or the psychiatric clinic will be done. If a participant discloses domestic violence, they will be informed about the support that is available to them.

The research database will be kept safely in a separate electronic device where only the researcher has access to the database. All data will be stored securely and confidentially, that is, hardcopy data in a locked cupboard in a secure room and softcopy data with password encryption.

Participant anonymity will be ensured in any future publications relevant to the study data.

### Analysis plan

The primary analyses will be based on complete cases only, excluding participants with missing data. We will also conduct a sensitivity analysis with all cases to explore whether this exclusion influenced our findings. All associations and descriptions will be stratified by sex. This is owing to the impact of domestic violence victimisation and perpetration being different in men and women and because suicidal behaviour is also different in men and women.

All analysis will be conducted using the STATA statistical software (V.18). We will report mean (SD) or median (interquartile range) for continuous variables and numbers and proportions for categorical variables. The associations between exposures and outcomes will be assessed using logistic regression models, and ORs with 95% CIs will be reported. The main outcome will be self-poisoning, and the exposures of interest will be domestic violence perpetration and victimisation. Three categories of domestic violence (ie, physical, emotional and sexual violence) will be considered. In the primary analysis, we will have two exposures of interest, and these will be dichotomised into a binary (yes/no) variable to indicate the presence of domestic violence perpetration and victimisation. For our secondary analysis, we will use a frequency score and we will calculate the frequency of each type of domestic violence. We will also investigate the potential effects of mental illness, harmful alcohol use, psychoactive substance use, income and ethnicity in a series of models.

### Strengths and limitations

A strength of this study is that it is one of the few studies to investigate the relationship between both domestic violence victimisation and/or perpetration and suicidal behaviour. This is an important but under-addressed area and, particularly, so in low and middle-income countries such as Sri Lanka.

Hospital-control outpatients may have a different exposure distribution from the general population, such as a greater prevalence of mood disorders and suicidal ideation, introducing the possibility of selection bias. We will mitigate this by purposively recruiting individuals who accompany patients and are less likely to be experiencing an acute health issue at the time of recruitment. Another possible limitation is that the reported rate of domestic violence (victimisation and perpetration) may be underestimated owing to cultural stigma and recall bias.

Participants may also downplay or deny perpetration of violence, owing to fear of retribution. To try to mitigate this, it will be clearly explained that information is being gathered for research purposes only, interviews will be conducted in a confidential space and trained data collectors will help create a comfortable environment by gradually introducing the topic and using permissive statements where appropriate. However, we recognise that confidentiality may have certain ethical and legal limitations. They will also be informed that there are exceptions where confidentiality may need to be broken—such as if there is an ongoing risk to a child or if a serious unreported crime is disclosed. In such cases, the principal investigator/senior team member will be available to assess the situation, and participants will be informed before any action is taken. Appropriate procedures will be followed based on the legal and ethical guidelines relevant to our study setting.

This study will focus on hospitalisations following self-poisoning; other forms of suicidal behaviour will not be investigated. But evidence shows that the vast majority of hospital-presenting self-harm in Sri Lanka is owing to self-poisoning.

### Ethics

Ethical approval for this research was obtained from the Ethical Review Committee of the Faculty of Medicine, University of Peradeniya, Sri Lanka. Further approval was obtained from the director of Teaching Hospital Paradeniya and the Consultant Physician of the Teaching Hospital Paradeniya’s toxicology unit to collect data from the subjects.

### Dissemination

The outcomes of this study will be disseminated through publication of findings in peer-reviewed articles. The results will also be shared at both national and international conferences, health symposia and local policy forums, such as those attended by local government and non-government organisations. We will prepare a short lay report with key study findings and will make it available to participants on request in local languages.

## References

[1] (2023). Suicide. https://www.who.int/news-room/fact-sheets/detail/suicide.

[2] (2021). Sri Lanka - suicide mortality rate (per 100,000 population) - 2021 data 2022 forecast 2000-2019 historical. https://tradingeconomics.com/sri-lanka/suicide-mortality-rate-per-100000-population-wb-data.html.

[3] De Silva V, Hanwella R, Senanayake M (2013). Age and sex specific suicide rates in Sri Lanka from 1995-2011. Sri Lanka J Psyc.

[4] Vijayakumar L (2015). Suicide in women. Indian J Psychiatry.

[5] Reid HE, Pratt D, Edge D Front psychiatry [internet]. 2022 mar 24 [cited 2023 dec 28];13. available from. Maternal Suicide Ideation and Behaviour During Pregnancy and the First Postpartum Year: A Systematic Review of Psychological and Psychosocial Risk Factors.

[6] de Brouwer AM, Kaitesi U (2016). Sexual violence. Elgar Companion to Int Crim Trib Rwanda.

[7] (2024). (PDF) intimate partner violence in sri lanka. Bioscience Trends.

[8] Correia CM, Gomes NP, Couto TM (2014). Representations about suicide of women with history of domestic violence and suicide attempt. Texto Contexto - Enferm.

[9] McManus S, Walby S, Barbosa EC (2022). Intimate partner violence, suicidality, and self-harm: a probability sample survey of the general population in England. Lancet Psychiatry.

[10] Amerio A, Aguglia A, Odone A (2020). Covid-19 pandemic impact on mental health of vulnerable populations. Acta Biomed.

[11] Knipe D, Vallis E, Kendall L Suicide Rates in High-Risk High-Harm Perpetrators of Domestic Abuse in England and Wales.

[12] Huntley AL, Potter L, Williamson E (2019). Help-seeking by male victims of domestic violence and abuse (DVA): a systematic review and qualitative evidence synthesis. BMJ Open.

[13] DCS (2019). Women ’ s wellbeing survey - 2019.

[14] Rajapakse TN (2017). A review of the changing patterns of suicide and deliberate self-harm in Sri Lanka. Sri Lanka J Psyc.

[15] Knipe D, Silva T, Aroos A (2021). Hospital presentations for self-poisoning during COVID-19 in Sri Lanka: an interrupted time-series analysis. Lancet Psychiatry.

[16] Bandara P, Page A, Senarathna L (2020). Domestic violence and self-poisoning in sri lanka. https://www.cambridge.org/core/journals/psychological-medicine/article/abs/domestic-violence-and-selfpoisoning-in-sri-lanka/621C6F4F2AFD0FB1D99ADE20BF464B14.

[17] Work with Perpetrators European Network (WWP-EN) (2015). Impact monitoring toolkit. https://www.work-with-perpetrators.eu/impact.

[18] Debt-finance-survey-qs-jan2022-v2- This questionnaire was developed by the research team, informed by a financial inclusion survey for Sri Lanka (Arandara, R. and Gunasekera, S. 2020, Women matter! Findings from Sri Lanka’s National Financial Inclusion Survey 2018/19, International Finance Corporation/Australian Aid) and survey questions developed to measure financial wellbeing in the UK (Kempson E., Poppe C. and Finney, A. 2017, Financial Well-Being A Conceptual Model and Preliminary Analysis, Norway: SIFO; Kempson, E. and Poppe, C. 2020, Coronavirus Financial Impact Tracker: Key findings from a national survey, April 2020, University of Bristol)..

[19] Hanwella R, Ekanayake S, de Silva VA (2014). The Validity and Reliability of the Sinhala Translation of the Patient Health Questionnaire (PHQ-9) and PHQ-2 Screener. Depress Res Treat.

[20] De Silva P, Jayawardana P, Pathmeswaran A (2008). Concurrent validity of the alcohol use disorders identification test (AUDIT). Alcohol Alcohol.

