# Correction: Domestic violence perpetration, victimisation and self-poisoning in Sri Lanka: a protocol for a hospital-based case-control study

**DOI:** 10.1136/bmjopen-2024-089913corr1

**Published:** 2025-04-30

 Hewa Kankanamge DV, Rubbo B, Morgan K, *et al*. Domestic violence perpetration, victimisation and self-poisoning in Sri Lanka: a protocol for a hospital-based case-control study. BMJ Open 2025;15:e089913. doi: 10.1136/bmjopen-2024-089913.

This article has been corrected since it was published online.

The license has been updated form CC BY NC to CC BY and the funding section has also been updated to:

This work was supported by the Elizabeth Blackwell Institute for Health Research (University of Bristol) and Wellcome Trust grant number [204813/Z/16/Z].

