# Supplementary material for: Domestic violence perpetration, victimisation and self-poisoning in Sri Lanka: a protocol for a hospital-based case-control study
Source: BMJ Open. 2025 Apr 3;15(4):e089913. doi: 10.1136/bmjopen-2024-089913 (PMC11969584; doi:10.1136/bmjopen-2024-089913)
Supplement: Supplementary file 1 [file bmjopen-2024-089913.reviewer_comments.pdf]

PEER REVIEW HISTORY

BMJ Open publishes all reviews undertaken for accepted manuscripts. Reviewers are asked to complete a checklist review form and are provided with free text boxes to elaborate on their assessment. These free text comments are reproduced below.

ARTICLE DETAILS

Title (Provisional)

Domestic violence perpetration, victimisation and self-poisoning in Sri Lanka: a protocol for a hospital-based case-control study

Authors

Hewa Kankanamge, Dilini Vidusha Vidusha; Rubbo, Bruna; Morgan, Karen; Cramer, Helen; Collard, Sharon; Ekanayaka, Ramya; Latheef, Afra; Athapaththu, Anushika; Siddeek, Mushfira; Wickramasuriya, Imalsha; Knipe, Duleeka W; Rajapakse, Thilini

---

VERSION 1 - REVIEW

**Reviewer**	**1**
**Name**	**Serafini, Gianluca**
**Affiliation**	**IRCCS Ospedale Policlinico San Martino**
**Date**	**02-Nov-2024**
**COI**	**None**

Thank you very much for asking me to review the present paper.

This is, in summary, an interesting hospital-based case-control study aimed to investigate the association between domestic violence (victimisation and perpetration) and self-poisoning in Sri Lanka. The authors reported that they will recruit a specific sample of subjects in order to test the association between suicide and victimization.

The authors may find as follows only my minor comments/suggestions.

First, as throughout the same section the authors correctly focused on the relevance of domestic violence, they might further stress that specific cases are at particular higher risk particularly following the Covid-19 pandemic in which an enhanced vulnerability to stressful situations emerged and still persist today. Thus, according to this background, the article published on Acta Biomed (PMID: 32701924) might be briefly cited within the main text.

Importantly, as the main aims/objectives of the present study have been correctly reported, the most relevant hypotheses might be similarly mentioned in a detailed manner.

In addition, the data collection and analysis plan could be provided more succinctly for the general readership.

Furthermore, the most relevant limitations/shortcomings of the present study need to be reported more extensively within the main text.

**Reviewer**	**2**
**Name**	**Naik-Mathuria, Bindi**
**Affiliation**	**University of Texas Medical Branch**
**Date**	**16-Dec-2024**
**COI**	**None**

The authors present a proposal of an interesting study to demonstrate the relationship between self-posioning and domestic violence (perpetration or victimization) in Sri Lanka, where both are prevalent. The planned study is a little confusing - case and control groups are clear, but it is unclear how the study staff will determine if the patient who tried to commit suicide perpetrated domestic violence (won't they keep this to themselves for fear of retribution/jail?). It may be more clear if the survey tools were included - all they say is that shortened versions of validated surveys will be used. There is a lot of additional social data that will be collected, but in it's current form it is unclear how the planned study will answer the research question.

VERSION 1 - AUTHOR RESPONSE

Reviewer: 1
Comments to the Author:
Thank you very much for asking me to review the present paper.

This is, in summary, an interesting hospital-based case-control study aimed to investigate the association between domestic violence (victimisation and perpetration) and self-poisoning in Sri Lanka. The authors reported that they will recruit a specific sample of subjects in order to test the association between suicide and victimization.

The authors may find as follows only my minor comments/suggestions.

1. First, as throughout the same section the authors correctly focused on the relevance of domestic violence, they might further stress that specific cases are at particular higher risk particularly following the Covid-19 pandemic in which an enhanced vulnerability to stressful situations emerged and still persist today. Thus, according to this background, the article published on Acta Biomed (PMID: 32701924) might be briefly cited within the main text.

We thank the reviewer for this suggestion, and we have cited the article in the Introduction section of the article.

2. Importantly, as the main aims/objectives of the present study have been correctly reported, the most relevant hypotheses might be similarly mentioned in a detailed manner.

Thank you for this suggestion. The hypothesis has been included as follows and it is included in the article.

'There is a positive association between experiences of domestic violence and self-harm. Specifically, individuals who have been subjected to or perpetrated domestic violence are hypothesised to have a higher risk of self-harm due to the psychological and emotional distress caused by such trauma (victims) or due to perceived victimhood or loss of control (perpetrators)'.

3. In addition, the data collection and analysis plan could be provided more succinctly for the general readership.

We sincerely appreciate the reviewer's insightful suggestion. In response, we have revised the data collection and analysis plan to present the information more succinctly, ensuring clarity and accessibility for the general readership. These modifications enhance the readability of our manuscript while maintaining the necessary detail. We believe these changes address the reviewer's concern and improve the overall presentation of our study.

4. Furthermore, the most relevant limitations/shortcomings of the present study need to be reported more extensively within the main text.
??

We thank the reviewer for making this important point. We have revised and discussed accordingly.

Reviewer: 2
Comments to the Author:
1. The authors present a proposal of an interesting study to demonstrate the relationship between self-posioning and domestic violence (perpetration or victimization) in Sri Lanka, where both are prevalent. The planned study is a little confusing - case and control groups are clear, but it is unclear how the study staff will determine if the patient who tried to commit suicide perpetrated domestic violence (won't they keep this to themselves for fear of retribution/jail?).

We thank the reviewer for their valuable comments. We acknowledge that some participants may deny or downplay their involvement in domestic violence due to fear of retribution. We have clarified this in the “strengths and limitations’ section and will take several steps to minimise this issue. All participants will be assured that the information collected is for research purposes only, interviews will be conducted in a confidential space, and trained data collectors will help create a comfortable environment by gradually introducing the topic and using permissive statements where appropriate.

However, we recognise that confidentiality may have certain ethical and legal limitations. While participants will be assured of confidentiality to encourage honest responses, they will also be informed that there are exceptions where confidentiality may need to be broken—such as if there is an ongoing risk to a child or if a serious unreported crime is disclosed. In such cases, the principal investigator/ senior team member will be available to assess the situation, and participants will be informed before any action is taken. We will follow appropriate procedures based on the legal and ethical guidelines relevant to our study setting.

2. It may be more clear if the survey tools were included - all they say is that shortened versions of validated surveys will be used.

Thank you, we have included the questionnaire as an appendix.

3. There is a lot of additional social data that will be collected, but in it's current form it is unclear how the planned study will answer the research question.

Thank you for your comment on this. The primary outcome of this study is suicidal behavior, and the main exposure is domestic violence, encompassing victimization and/or perpetration. The additional social data collected, data on socio-economic indicators such as occupation, education level, and vehicle ownership will help to assess socio-economic status, which could act as a confounder in the relationship between domestic violence and suicidal behavior. Similarly, depression (measured via PHQ-9), harmful alcohol use (AUDIT), psychoactive substance use, income, and ethnicity will be incorporated into a series of models to examine their roles as potential mediators or moderators.

By integrating these variables into our analysis, we aim to account for the complex interplay of factors that influence suicidal behavior and its association with domestic violence. This approach ensures that the study design is comprehensive and capable of addressing the research question.
